# 5-Aminolevulinic acid treatment mitigates pesticide stress in bean seedlings by regulating stress-related gene expression and retrotransposon movements

**DOI:** 10.1007/s00709-023-01924-9

**Published:** 2024-01-08

**Authors:** Esra Arslan Yuksel, Murat Aydin, Guleray Agar, Mahmut Sinan Taspinar

**Affiliations:** 1 Faculty of Agriculture, Department of Agricultural Biotechnology, Ataturk University, 25240 Erzurum, Turkey; 2 Faculty of Science, Department of Biology, Ataturk University, 25240 Erzurum, Turkey

**Keywords:** ALA, Antioxidant genes, Deltamethrin, QPCR, REMAP, SAP gene

## Abstract

Overdoses of pesticides lead to a decrease in the yield and quality of plants, such as beans. The unconscious use of deltamethrin, one of the synthetic insecticides, increases the amount of reactive oxygen species (ROS) by causing oxidative stress in plants. In this case, plants tolerate stress by activating the antioxidant defense mechanism and many genes. 5-Aminolevulinic acid (ALA) improves tolerance to stress by acting exogenously in low doses. There are many gene families that are effective in the regulation of this mechanism. In addition, one of the response mechanisms at the molecular level against environmental stressors in plants is retrotransposon movement. In this study, the expression levels of superoxide dismutase (SOD), ascorbate peroxidase (APX), catalase (CAT), glutathione reductase (GR), and stress-associated protein (SAP) genes were determined by Q-PCR in deltamethrin (0.5 ppm) and various doses (20, 40, and 80 mg/l) of ALA-treated bean seedlings. In addition, one of the response mechanisms at the molecular level against environmental stressors in plants is retrotransposon movement. It was determined that deltamethrin increased the expression of SOD (1.8-fold), GPX (1.4-fold), CAT (2.7-fold), and SAP (2.5-fold) genes, while 20 and 40 mg/l ALA gradually increased the expression of these genes at levels close to control, but 80 mg/l ALA increased the expression of these genes almost to the same level as deltamethrin (2.1-fold, 1.4-fold, 2.6-fold, and 2.6-fold in SOD, GPX, CAT, and SAP genes, respectively). In addition, retrotransposon-microsatellite amplified polymorphism (REMAP) was performed to determine the polymorphism caused by retrotransposon movements. While deltamethrin treatment has caused a decrease in genomic template stability (GTS) (27%), ALA treatments have prevented this decline. At doses of 20, 40, and 80 mg/L of ALA treatments, the GTS ratios were determined to be 96.8%, 74.6%, and 58.7%, respectively. Collectively, these findings demonstrated that ALA has the utility of alleviating pesticide stress effects on beans.

## Introduction

Dry bean is a warm-season plant that is rich in protein and vitamins and can easily grow in almost any type of soil. Also, it has a large cultivation area and is an important legume in terms of production (28.9 million tons, FAO 2019) among edible legumes in the world. However, the richness of the bean’s protein ratio increases its susceptibility to diseases and harmful insects (Mullins and Arjmandi 2021). In this sense, it becomes necessary to use modern agricultural techniques and inputs in order to increase the yield and quality of agricultural products and to combat diseases and pests.

The use of pesticides is a form of agricultural struggle in order to protect agricultural products from the damage of diseases, pests, and weeds. Deltamethrin [(S)-α-cyano-3-phenoxybenzyl (1R, 3R)-cis-2,2-dimethyl-3- (2,2-dibromovinyl)-2,2-cyclopropanecarboxylate] is a synthetic pyrethiroid and a broad-spectrum insecticide (Sayeed et al. 2003). It is known that deltametrin has been used successfully to control aphid infestation in fields where important crops such as beans are grown (Johnstone 1984). However, many sections of plants, such as cells (Mukhopadhyay et al. 2006), genomes (Chauhan et al. 2007; Ansari et al. 2009; Aylward et al. 2011), and chromosomes (Marques et al. 2014), are negatively affected due to the accumulation and non-degradation of insecticides by forming insoluble bonds in agricultural products (Bashir et al. 2007). In this case, many genes are activated to protect crops from the effects of pesticide stress (Kishimoto et al. 2002; Tian et al. 2013). The expression of antioxidant enzyme genes [superoxide dismutase (SOD), ascorbate peroxidase (APX), catalase (CAT), and glutathione reductase (GR)] increases in order to avoid the harmful effects of ROS, whose amount increases against oxidative stress that occurs during pesticide stress. Furthermore, stress-associated proteins (SAPs) are known as response factors to abiotic and biotic stresses and confer stress tolerance to plants (Wang et al. 2020).

One of the response mechanisms created at the molecular level against environmental stressors is retrotransposon movement in plants. Although they are inactive during normal growth and development, they are activated during stress, increase the mutation rate, and also cause methylation changes in the genome. It has been determined that Ttd1a retrotransposon activated when exposed to salt and light stress, located next to a resistance gene, and thus protected the wheat against stress (Woodrow et al. 2010). Retrotransposon-based markers have a key role in determining retrotransposon movements induced by stress. REMAP is one of these markers that amplify the DNA region between retrotransposon and simple sequence repeats (Kalendar et al. 2011).

Plants activate many plant growth regulators (PGRs) as well as antioxidant defense mechanisms, genes, and retrotransposon movements in order to tolerate damage to their metabolism. Also, the exogenous use of PGRs has a positive effect on increasing stress tolerance in stressed plants (Ali et al. 2013). One of these regulators, 5-aminolevulinic acid (ALA), is a precursor molecule involved in the biosynthesis of porphyrins such as chlorophyll (Chl), vitamin B12, and heme in plants (Balestrasse et al. 2010; Ali et al. 2013). The exogenous application of ALA is an effective antistress agent under optimum conditions that plays a role in the development of plant tolerance. It has been demonstrated in many stress studies, such as low temperature (Balestrasse et al. 2010), high temperature (Zhang et al. 2012), low light (Sun et al. 2009), excessive salinity (Naeem et al. 2011), heavy metal stress (Ali et al. 2013), and herbicide stress (Zhang et al. 2008).

In this study, we aimed to determine the expression levels of the SOD, CAT, GPX, and SAP genes, which are induced by the activation of the antioxidant mechanism against the oxidative damage caused by deltamethrin when used in excessive doses. Furthermore, it was assessed whether ALA, which has previously been shown to have a healing role in our earlier study (Taspinar et al. 2017), induced a change in the expression of these genes when combined with deltamethrin. Also, retrotransposon mobility and the rate of polymorphism were determined using the REMAP technique.

## Material and methods

### Plant material

*Phaseolus vulgaris* L. cv. Elkoca seeds were used as plant material provided by the Ataturk University Faculty of Agriculture.

### Growth conditions, ALA, and deltamethrin treatments

The seeds used in the experiment were selected based on their equal sizes, sterilized with a 5% hypochlorite solution for 5 min, and rinsed three times with distilled water. Then, they were germinated in a hydroponic system at 25 °C for 16 light and 8 dark hours in plastic boxes (Arslan 2021) containing Hoagland solution (Sigma H2395-10 L) (Hoagland and Arnon 1938). By selecting from 7-day-old plants, 10 seedlings were obtained in an equivalent growth time. These seedlings were kept in the same conditions as the others. The experiment was conducted using a completely random design with three replications. ALA solutions (Sigma, A3785) [0 (control), 20, 40, and 80 mg/l] were sprayed on 20-day-old seedling leaves (Beyzaei et al. 2015). After 5 days of ALA treatment, a 0.5 ppm deltamethrin solution (Sigma, 45423) was sprayed on the leaves (Duran et al. 2015). Bulk sample strategy was applied for molecular analysis. Leaf samples were harvested 5 days after deltamethrin application from five randomly selected plants for each replication of treatments and were stored at − 80 °C.

### Total RNA extraction, cDNA synthesis, and gene expression

Total RNA from 100 mg of leaves was extracted with the RNeasy Plant Mini Kit (Qiagen) according to the suppliers’ instructions. RNA purity and concentrations were assessed by determining the spectrophotometric absorbance of the samples with a NanoDrop-1000 spectrophotometer (OD 260/230>2). RNA integrity was evaluated on a 1.2% agarose gel, stained with ethidium bromide, and visualized with UV light. First-strand cDNA synthesis was performed with the RevertAid First-Strand cDNA Synthesis Kit (Thermo Scientific) as described by Arslan et al. (2021). The quantitative PCR was performed with the SYBR Green/ROX qPCR Kit (Thermo Scientific) according to the manufacturer’s protocol, and the following genes were amplified: SOD, GPX, CAT, and SAP. β-Actin was preferred as a housekeeping gene. To design the primers for genes, databases related to bioinformatic studies conducted in the Phaseolus vulgaris genome were used (Table 1). Accession numbers for genes were found using the Pfam (pfam.xfam.org) database. Then, the Phytozom (https://phytozome.jgi.doe.gov/) database, which is the plant genomic source, was used. Finally, primers were created using the Primer3 (http://frodo.wi.mit.edu/) program from selected base sequences. The Q-PCR reactions were run in a Qiagen Rotor-Gene, and the cycling conditions consisted of initial denaturation at 95 °C for 10 min, followed by 40 cycles of amplification at 95 °C for 15 s, 56–65 °C for 30 s, and extension at 72 °C for 30 s. The relative gene expression levels, which were determined using the 2^−ΔΔCt^ equation, were calculated to get the expression fold change (Livak and Schmittgen 2001). Each sample was analyzed in three technical replicates. A one-way ANOVA was performed to evaluate the effect of treatments on gene expression. Duncan’s multiple range test (*P* ≤ 0.05) was performed to compare the mean values. The data were analyzed using SAS 9.3 software for Windows.

**Table 1:** Q-PCR primer sequences used in the present study

Targeted gene	Primers	Sequence (5′-3′)	Annealing temperature (°C)
SOD	**F**	TCACAGGGAGAATAACAGGGT	60.0
	**R**	ACCTGCATTCCCAGTAGTCT	61.0
CAT	**F**	AACTTCCCCGTCTTCTTCATC	58.0
	**R**	GTTGTTCTCCTTCTCGATCACC	56.0
GPX	**F**	GCAGATACAAGGGGAAAGTCC	56.0
	**R**	CCAACAGCTTCTTGATGTCATT	56.0
SAP	**F**	CGAGTTCAAGGTTCCCGAAA	62.0
	**R**	GTCGTAACTGCAGTCGTGG	60.0
β-Actin	**F**	CCATCAAGACCAAGCG	60.0
	**R**	GTCAATGCGGGAGAAG	60.0

### Genomic DNA extraction

Total DNA was extracted from 0.1 g of leaves from each treated group by the CTAB method of Shams et al. (2020). Integrity and quality of DNA were evaluated by electrophoresis on a 1% agarose gel.

### REMAP

The REMAP reactions were based on a previously published method (Yigider et al. 2020). For amplification, the IRAP primers [Nikita-E2647, Stowaway, Sukkula, and Bare 1(0)] were combined with ISSR primers (8081, 8082) (Table 2). Amplifications were carried out in a Thermo Scientific™ Arktik™ Thermal Cycler with the following PCR programs: an initial denaturation at 95 °C for 5 min, followed by 35 cycles of 94 °C for 1 min, distinct temperatures for each primer for 1 min, and 72 °C for 2 min; and a final extension at 72 °C for 15 min. PCR products were run by electrophoresis on 2% agarose gel in 0.5× TBE buffer stained with ethidium bromide and photographed in the DNR Minibis Gel Documentation System (USA).

**Table 2:** Name and sequence of primers used in the present study

ISSR		Retrotransposons	
Primer	Sequence (5′-3′)	Primer	Sequence (5′-3′)
8081	(GA)_9_C	Nikita-E2647	ACCCCTCTAGGCGACATCC
8082	(CT)_9_G	Stowaway	CTTATATTTAGGAACGGAGGGAGT
		Sukkula	GATAGGGTCGCATCTTGGGCGTGAC
		Bare 1(0)	CTAGGGCATAATTCCAACA

### REMAP data analysis

The gel images obtained were evaluated with the TotalLab TL120 program. Genomic template stability (GTS) (%) for each primer was calculated using the formula 100 − (100 × *a*/*n*) according to Atienzar et al. (1999). “*a*” in the formula indicates the REMAP polymorphic profiles determined for each treated sample, and “*n*” indicates the total number of DNA bands obtained in the negative control group with the relevant primer. The polymorphism observed in the REMAP profiles of the treatment groups includes the emergence of a new band or the disappearance of an existing band compared to the negative control group. For all treatments, a binary matrix was generated based scored as 1 (present) or 0 (absent) for each primer. The following calculations were carried out with the use of NTSYSpc 2.11f software. The Jaccard’s similarity coefficient was calculated by using the SIMQUAL module. The similarity coefficients were then used to construct dendrograms, by using the UPGMA (unweighted pair group method with arithmetic averages) employing the SAHN. The goodness of dendongram was verified in the MXCOMP program by using Jacard’s similarity matrix and co-phenetic value matrix. The three-dimensional PCoA was performed based on the similarity matrix.

## Results and discussion

### Gene expression profile of some antioxidant enzymes and stress protein under deltamethrin and ALA treatments

Unfavorable environmental conditions and insect infestations are the strongest factors limiting yield in beans (Gogo et al. 2014). Pesticides, such as deltamethrin, are frequently used in agricultural lands to reduce the effects of harmful organisms. Overdose of deltamethrin causes oxidative damage in plants by activating ROS (Bashir et al. 2007). ROS play a dual role in plant responses to abiotic stress, both as toxic by-products of stress metabolism and as an important signal transduction molecule in complex metabolic processes responsible for the emergence of stress responses based on calcium, hormone, and protein phosphorylation (Miller et al. 2010). The uncontrolled oxidation obtained when ROS are overproduced leads to cellular damage and ultimately cell death. In order to prevent the plant from being damaged by this situation, the current antioxidant mechanism should keep active oxygen under control (Bose et al. 2014). When these removal mechanisms are not damaged by stress, ROS are rapidly destroyed by antioxidant mechanisms (Ahmad et al. 2010). Among the regions targeted by ROS, such as proteins and DNA, that are difficult to repair result in genetic damage. Genetic studies in seedlings of *Phaseolus vulgaris* also suggest a link between pesticide stress and oxidative stress, since deltamethrin induces the expression of several genes that are also induced by oxidative stress (Ajermoun et al. 2022; Boulahia et al. 2023). In this study, expression levels of the SOD, CAT, and GPX genes, which are antioxidant genes, were determined. According to our results, all of these genes were upregulated by deltamethrin stress.

**Fig. 1:**
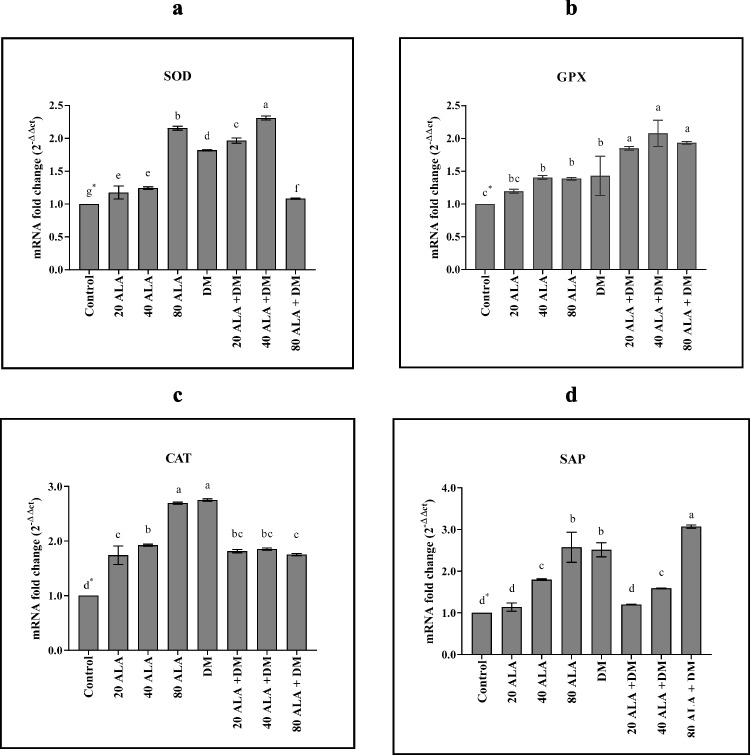
The expression patterns of **a** SOD, **b** GPX, **c** CAT, and **d** SAP genes under 5-aminolevulinic acid (ALA) and deltamethrin (DM) treatments. Data represent the means ±SD of three replications. The different letter on the graph indicates significant differences based on Duncan’s multiple range test (*P* ≤ 0.05)

SOD is the first line of defense against ROS during abiotic stress in a plant cell. Control of SODs in both expression and activity of ROS contributes to the regulation of stress tolerance (Forman 2007; Liu et al. 2008). According to the results of the SOD gene expression analysis, the rate of gene expression in deltamethrin treatment alone was about twofold higher than that of the control (1.8) (Fig. 1a). Similarly, GPX and CAT genes were upregulated in deltamethrin treatment approximately two and threefold (1.4 and 2.7) according to non-treated seedlings, respectively (Fig. 1b, c). To scavenge the ROS efficiently, the activity of APX and SOD must be high to remove the H_2_O_2_ produced by superoxide ion dismutation (Pospíšil 2012). Therefore, in our study, the high expression levels of both SOD, CAT, and GPX genes could be responsible for the removal of ROS. In a similar study, Sharma et al. (2015) investigated the expression levels of CAT, SOD, APX, and GR enzyme genes in salt and pesticide stress applied to rice and found that all genes were highly upregulated in both stresses.

As well as antioxidant genes, the SAP gene was highly upregulated (2.5) in deltamethrin stress (Fig. 1d). Proteins in the SAP family contain the A20/AN1 zinc finger domain and are known to be important determinants of stress responses in plants (Vij and Tyagi 2006). Similar results were obtained in different plants during different abiotic stresses, indicating that SAP genes (OSISAP) found in rice in particular are induced by abiotic stress (Vij and Tyagi 2006). Overexpression of the OSISAP1 gene in tobacco (Nicotiana tabacum) increases tolerance to cold, drought, and salt stress (Mukhopadhyay et al. 2004). Similarly, the OSISAP8 gene, which is transferred to the tobacco plant, is thought to have a role in the development of tolerance against abiotic stress (Kanneganti and Gupta 2008). Giri et al. (2011) determined that the OSISAP11 gene transferred to transgenic Arabidopsis interacts with OSIRLCK253, a receptor-like cytoplasmic kinase, providing tolerance to drought and salt stress, as well as the TaSAP5 gene in wheat (Zhang et al. 2017). Studies have associated SAP proteins with roles such as ubiquitination, redox detection, and regulation of gene expression under abiotic stress (Ströher et al. 2009; Ben-Saad et al. 2012; Kang et al. 2013). However, the mechanism by which SAP proteins play the main role mechanically in stress responses has not been fully elucidated.

In recent studies, plant growth regulators have also been reported to have roles in the regulation of the plant defense system against various stresses (Zhang et al. 2008; Balestrasse et al. 2010; Naeem et al. 2011; Zhang et al. 2012; Ali et al. 2013). In addition, it has been proven that high concentrations of ALA play a role as an herbicide or insecticide (Chon 2003). As a matter of fact, in our study, ALA in low doses (20 and 40 mg/l) was beneficial in creating stress tolerance by increasing the expression of antioxidant genes. In the 80 mg/l ALA application, it was determined that SOD gene expression was higher than in the delthametrin treatment, and the difference between these two treatments had been significant (Fig. 1a). On the other hand, CAT and GPX gene expressions in 80 mg/l ALA application were close to the results obtained from deltamethrin application (Fig. 1b, c). In addition, when ALA and deltamethrin were treated together, 40 mg/l of ALA increased the expression of these genes more. This situation may be related to ALA protecting the cell against the destructive effect of ROS by removing H_2_O_2_ (Ali et al. 2015). Sharma et al. (2015) found that the SOD gene was more induced in brassinosteroid applications than salt or pesticide stress alone in rice. Exogenously applied high concentrations of ALA accumulate in excessive amounts in cells, causing an increase in the amount of ROS by being exposed to both enolization and aerobic oxidation with metal catalysis (Reyter and Tyrrel 2000). In this case, the enzyme called heme oxygenase degrades the free heme group, converts bilirubin into iron and carbon monoxide (Shekhawat and Verma 2010), and causes a decrease in oxidative stress in the plant (Grochot-Przeczek et al. 2012). While the mechanism for oxidative stress and degradation of the heme group remains unclear, it is thought to be an evolutionary protection mechanism given by the plant cell to counteract the destructive effect of the free heme group (Kumar and Bandyopadhyay 2005). Noriega et al. (2012) determined that the cadmium increased the ALA content in the root, leaf, and nodule parts of the soybean, and the plant was exposed to more oxidative stress. At the same time, it was found that cadmium or ALA applications both inhibited antioxidant enzyme activities and caused a significant decrease in SOD and guaicole peroxidase expression.

Our experiment results also indicated that SAP gene was gradually upregulated in ALA treatments. However, the highest expression rate was 80 mg/l ALA + deltamethrin and the lowest was in the application of 20 mg/l ALA. ZFP185, a A20/AN1 zinc finger protein, is linked to abscisic acid and gibberellic acid, which regulate the cell growth and stress response mechanism (Zhang et al. 2016). Thus, it can be assumed that the SAP gene works in conjunction with these hormones to establish stress tolerance. In our study, it is thought that 80 mg/l ALA application has a more damaging effect on deltamethrin by acting as an insecticide; it may have a role in the formation of stress tolerance by linking with signal molecules such as abscisic acid and gibberellic acid, which leads to a greater increase in the expression of these genes.

### Changes in REMAP pattern under deltamethrin and ALA treatments

Another effect of various environmental stressors at the genome level is retrotransposon mobility. In our study, the retrotransposon polymorphism caused by deltamethrin was determined by REMAP. A total of eight primer pairs were tried for REMAP analysis, and 113 bands were obtained, and 98 of them were determined to be polymorphic bands. All of the primers were determined to be polymorphic. Maximum number of polymorphic bands counted in Bare 1 (0) + ISSR 8081 and minimum in Stowaway + ISSR 8081 primer pairs (Table 3). The polymorphic information content (PIC) value of the primer pairs used to determine the molecular effects of the treatments varied between 0.365 and 0.427, and the average was 0.382 (Table 3). The maximum PIC value for dominant markers is 0.5. Because two alleles are assumed per locus, both are affected by the number and frequency of alleles. In this respect, the Stowaway + ISSR 8081 primer pair had the highest PIC value (Table 3). On the other hand, the discriminating power (*D*) parameter used in the evaluation of the primers shows the efficiency of the primers in the identification of individuals. The *D* value of the primers varied between 0.408 and 0.867 (Table 3). The Bare 1 (0) + ISSR 8081 primer pair, which has both the discrimination power and the highest polymorphic band content, was determined to be the most distinctive primers.

**Table 3:** Polymorphic information content, discrimination power, and polymorphic band counts of primer pairs used in REMAP analysis

Primers	Polymorphic information content (PIC)^a^	Discrimination power (*D*)^b^	Polymorphic band counts/total band counts
Nikita-E2647 + ISSR 8081	0.365	0.776	14/15
Nikita-E2647 + ISSR 8082	0.374	0.642	13/15
Stowaway + ISSR 8081	0.427	0.408	7/12
Stowaway + ISSR 8082	0.372	0.658	13/13
Sukkukla + ISSR 8081	0.373	0.652	13/15
Sukkula + ISSR 8082	0.369	0.676	12/14
Bare 1(0) + ISSR 8081	0.381	0.867	16/16
Bare 1(0) + ISSR 8082	0.399	0.523	10/13
Average	0.382	0.650	-
Total			98/113

^a^Botstein et al. (1980)

^b^Prevost and Wilkinson (1999)

**Fig. 2:**
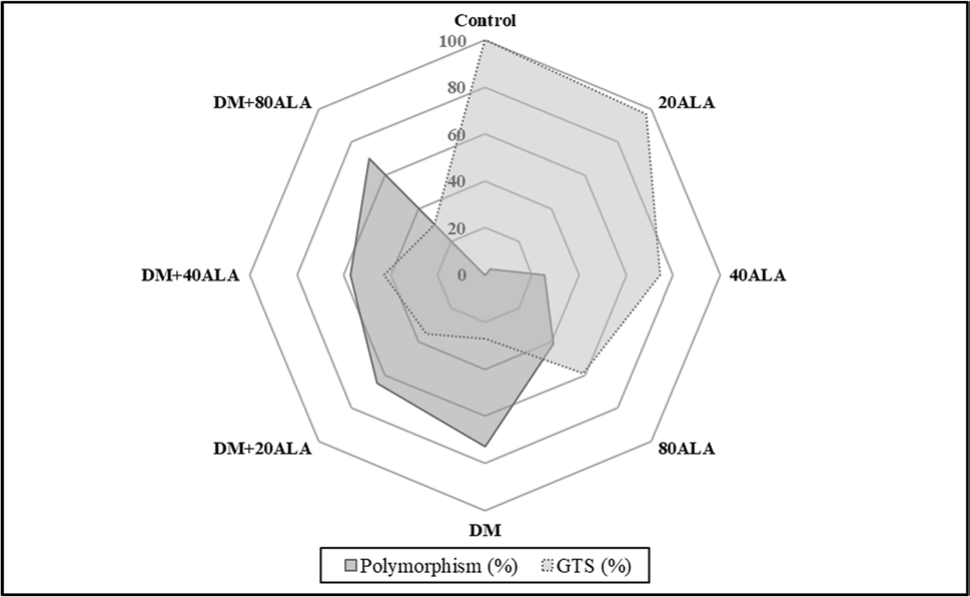
Changes in GTS and polymorphism value under 5-aminolevulinic acid (ALA) and deltamethrin (DM) treatments

With the treatment of deltamethrin, a 73% polymorphism ratio occurred. When examining the effects of applications in terms of GTS ratio, the lowest GTS value was obtained in deltamethrin-treated seedlings compared to the control. While the molecular weights of the missing bands of applications compared to the control are between 50 and 1440 bp, the newly formed bands were between 224 and 1581 bp (Table 4). When the effects of different doses of ALA were examined compared to the control, it was determined that 40 and 80 mg/L ALA doses caused a decrease in the GTS values, depending on the dose increase. On the other hand, 40 mg/L ALA reduced the negative effect of deltamethrin on GTS (Fig. 2).

**Table 4:** Molecular weights (bp) of new (+) and disappearing (−) bands occurring in 5-aminolevulinic acid (ALA) and deltamethrin (DM) treatments according to control

Primers	Band count (control)	+/−	0.5 ppm DM	0 ppm DM			0.5 ppm DM		
				20 mg/L ALA	40 mg/L ALA	80 mg/L ALA	20 mg/L ALA	40 mg/L ALA	80 mg/L ALA
Nikita –E2647 + ISSR 8081	7	**+**	246 515 709 891	444	246 515 667 709	143 246 444 515	143 444 515 709	143 515	444 515 709 891 1018
		**-**	808 305 209 94	808 396	808 305 209 94	573 396 209 94	573 396 209	-573 -396 -209	-808 -573 -396 -209
Nikita-E2647 + ISSR 8082	11	**+**	309 400 733	-	733	465	733	465	309 733
		**-**	1440 1161 1049 795 362 50	-	1440 795 362 267 50	504 267	1440 1161 1049 795 362	504	1440 795 621 362 267 50
Stowaway + ISSR 8081	9	**+**	430 1047	-	430 464 1047	430	430 1047	430	430 464 1047
		**-**	350	-	393	608 350	350	832 608 393 350	393
Stowaway + ISSR 8082	8	**+**	347 411	347	411 532 781	256 411 532	411 532 781	411 532	532 781
		**-**	459 390 314 247	-	741 599 459 390 247	503 390	686 503 390 247	503	741 503 247
Sukkula + ISSR 8081	8	**+**	114 571 1450 1581	980	1209 1450	465 571 1209 1450	114 571 1450	114 465 571 980	571 1209
		**-**	504 136	-	1279 1127 504	1127 504	136	1127 917 504 95	504
Sukkula + ISSR 8082	7	**+**	663 836 1004 1455	836	756 862 1004	862 1455	663 836	454 663	454 663 836 862
		**-**	1092 709	709 540	615	1092 540	792	615	709
Bare 1(0) + ISSR 8081	5	**+**	792	443 792 824 902 1024 1200	248 282 290 443 792	393 902	290 393	393	154 248 443
		**-**	691 214	340 214	691 340	691 340	691 340	603	691 486
Bare 1(0) + ISSR 8082	8	**+**	224 624 702	258 702	224 306 624	224 258 702	224 258 306 702	224 258 306 624 702	224 258 702
		**-**	787 658	658 286	787 449	890	787 449 286	890 658 286	787 286
GTS (%)			27	96.8	74.6	58.7	35	42.9	30.2
Polymorphism (%)			73	3.2	25.4	41.3	65	57.1	69.8

**Fig. 3:**
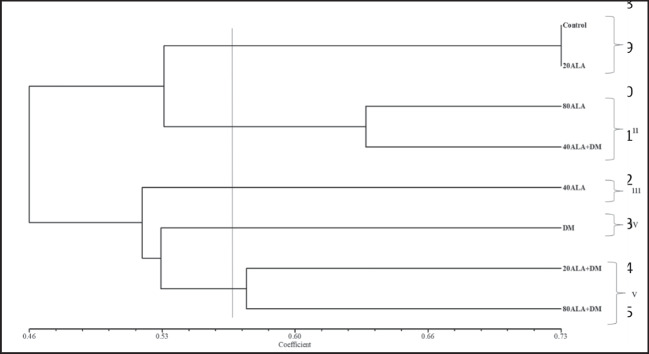
The dendrogram obtained from REMAP data using Jaccard’s coefficients of similarity and UPGMA clustering

**Fig. 4:**
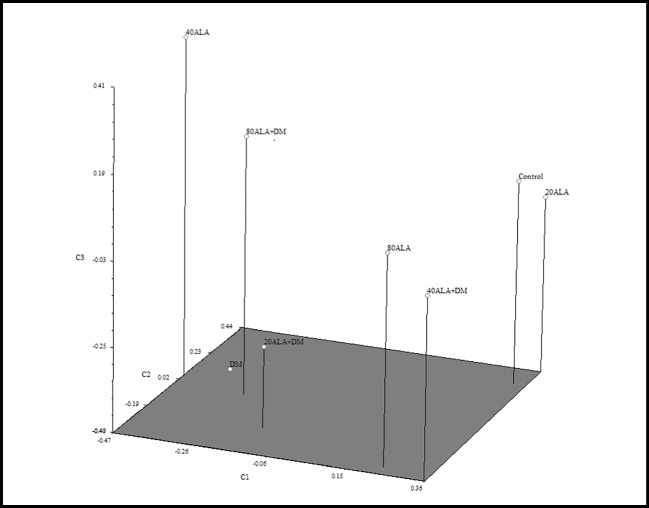
Distribution of treatments by three-dimensional principal coordinate analysis using Jaccard’s similarity

The similarity index of the applications varied between 0.38 and 0.73. The highest similarity with the control occurred between 20 mg/L ALA, while the least similarity was deltamethrin treatment (Table 5). This result showed that deltamethrin caused a significant change induced by retrotransposons in the genome. On the other hand, when the similarity indexes of ALA applications against deltamethrin applications with control were evaluated, it was determined that 40 mg/L ALA applications against deltamethrin had the highest similarity between these applications with control (Table 5). Furthermore, genetic similarity values were used for cluster analysis through UPGMA, resulting in a dendrogram (Fig. 3). The cophenetic correlation coefficient was calculated to evaluate the goodness of the dedongram. This value was determined as 0.84 and indicated a good fit (Rholf 1993). The UPGMA analysis clearly indicated differences among treatments. The treatments were grouped into five clusters, with cluster I containng control and 20 mg/L ALA, cluster II containing 80 mg/L ALA and 80 mg/L ALA + DM, cluster III containing 40 mg/L ALA, cluster IV containing DM, and cluster V containing 20 mg/l ALA + DM and 80 mg/L ALA + DM (Fig. 3). The results of PCoA support the results obtained from cluster analysis obtained through UPGMA (Fig. 4). While there are many studies on increasing retrotransposon mobility and polymorphism with stress, there is no literature about deltamethrin stress. Evrensel et al. (2011) investigated the mobility of Nikita and BARE-1 retrotransposons in barley (*Hordeum vulgare* L.) under plant tissue culture conditions using the IRAP molecular marker technique and reported that the polymorphism that occurs in callus of different ages is due to the movements of Nikita and BARE-1 retrotransposons. Yigider et al. (2020) determined the polymorphism resulting from the movement of some retrotransposons by heavy metal stress in maize using IRAP and REMAP techniques. In our previous study, where we determined the effect of deltamethrin and ALA applications on DNA methylation changes (Taspinar et al. 2017), the high level of DNA methylation polymorphism caused by deltamethrin decreased to lower values with ALA. Furthermore, a change in the GTS rate was observed at all doses of ALA in this study. This may be due to epigenetic change. Taspinar et al. (2017) indicated that ALA caused changes in DNA methylation in *Phaseolus vulgaris*. Reinders et al. (2009) and Mirouze and Paszkowski (2011) reported that epigenetic situation changes may promote the movement of DNA transposons and retroelements, which are abundant in the plant genome.

**Table 5:** Matrix of pairwise genetic similarity between 5-aminolevulinic acid (ALA) and deltamethrin (DM) treatments based on Jaccard’s coefficients

	Control	20ALA	40ALA	80ALA	DM	20ALA + DM	40ALA + DM	80ALA + DM
Control	1.000							
20ALA	0.733	1.000						
40ALA	0.460	0.379	1.000					
80ALA	0.554	0.511	0.444	1.000				
DM	0.465	0.444	0.512	0.433	1.000			
20ALA + DM	0.512	0.472	0.488	0.548	0.549	1.000		
40ALA + DM	0.556	0.494	0.366	0.633	0.385	0.530	1.000	
80ALA + DM	0.489	0.484	0.553	0.489	0.506	0.571	0.456	1.000

## Conclusion

Overall, our results unequivocally established that SOD, CAT, GPX, and SAP genes are induced by the activation of the antioxidant mechanism against the oxidative damage caused by deltamethrin. In addition, it was determined that ALA caused a change in the expression of these genes when applied together with deltamethrin. Thus, an important step of the plant’s response mechanism against stress has been elucidated. At the same time, the retrotransposon mobility caused by deltamethrin stress and the effect of ALA on this mobility and its polymorphism ratio were determined using the REMAP technique. In this respect, it is thought that this is the first study conducted on this subject, and the results obtained as a result of this study will lead to the cultivation of the bean plant, which has an important place in the world, on lands exposed to intense pesticide stress and lead to other studies in this field.
